# Medial circumflex femoral artery flap for ischial pressure sore

**DOI:** 10.4103/0970-0358.45028

**Published:** 2009

**Authors:** S. Palanivelu

**Affiliations:** Department of Plastic Surgery, Coimbatore Medical College Hospital, Coimbatore - 18, India

**Keywords:** Ischial pressure sore, Medial circumflex femoral artery, flap, Ischial sore

## Abstract

A new axial pattern flap based on the terminal branches of the medial circumflex femoral artery is described for coverage of ischial pressure sore. Based on the terminal branches of the transverse branch of medial circumflex femoral artery, which exit through the gap between the quadratus femoris muscle above and the upper border of adductor magnus muscle below, this fascio cutaneous flap is much smaller than the posterior thigh flap but extremely useful to cover ischeal pressure sores. The skin redundancy below the gluteal fold allows a primary closure of the donor defect. It can also be used in combination with biceps femoris muscle flap.

## INTRODUCTION

There are many flaps described for coverage of ischial pressure sores. Gluteus maximus myocutaneous flap, medially based posterior thigh flap, inferior gluteal artery island flap and V-Y advancement of hamstring muscles are some of the commonly employed flaps for coverage. Medially based posterior thigh flap is a large random pattern skin flap occupying almost the upper two-thirds of the posterior aspect of the thigh with a wide pedicle based on the medial side of the thigh.[1] On transposition it leaves a secondary defect below, which has to be covered with split-skin graft. We describe a much smaller fasciocutaneous flap which may be based either medially or laterally just below the gluteal fold for coverage of ischial pressure sore. This flap is an axial pattern flap supplied by the terminal branches of the medial circumflex femoral artery.

### Vascular basis of the flap

Four arteries anastamose below the gluteal fold in a deep intermuscular plane to form the cruciate anastomosis. They are the terminal branches of medial circumflex femoral artery, terminal branches of lateral circumflex femoral artery from medial and lateral side respectively, descending branch of inferior gluteal artery from above and first perforating branch of profunda femoris artery from below. [2] Of these arteries the terminal branches of the transverse branch of medial circumflex femoral artery exit through the interval between quadratus femoris muscle above and the upper border of adductor magnus muscle below to reach the deep fascia of the thigh.[2] A fasciocutaneous flap based either medially or laterally, raised carefully, preserving the perforators arising from the medial circumflex femoral artery will be a very safe flap. A much smaller flap than the posterior thigh flap may be used. In addition we may take advantage of the redundancy of skin below the gluteal fold to primarily close the secondary defect resulting from transposition of the flap.

## MATERIAL AND METHODS

**Figure 1:**
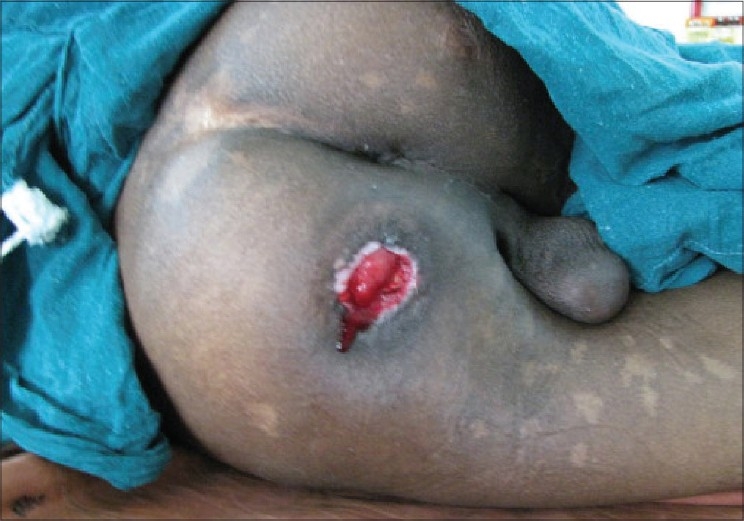
Ischial pressure sore

**Figure 2:**
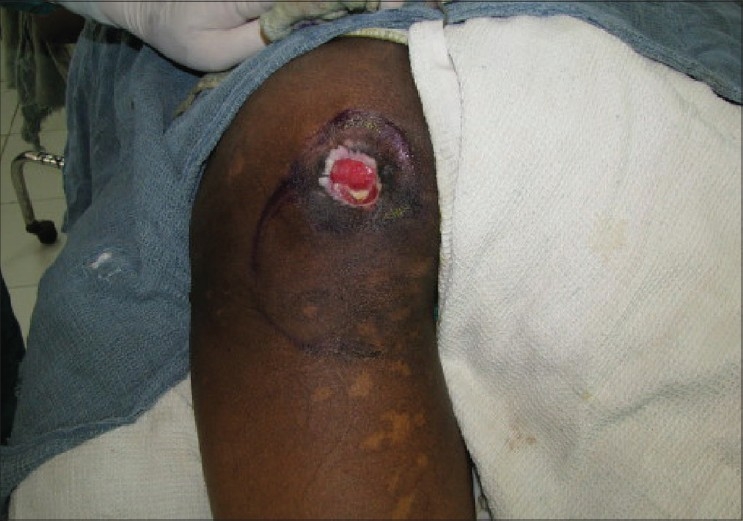
Flap marked before sore excision

**Figure 3:**
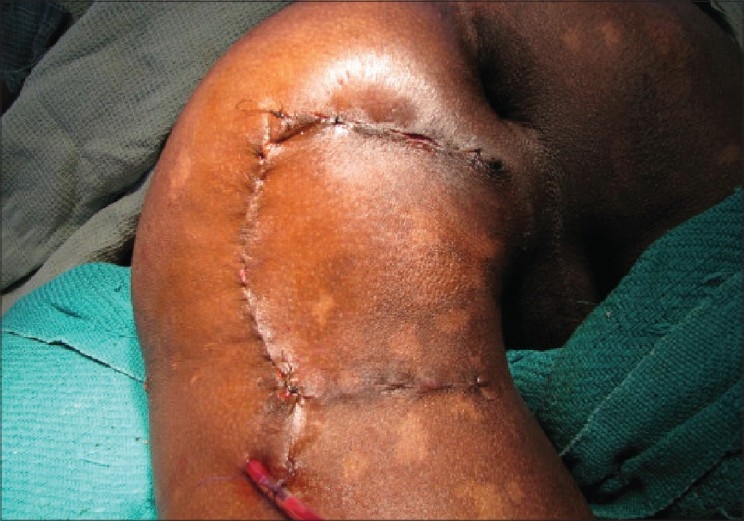
Flap transposed and secondary defect closed

**Figure 4:**
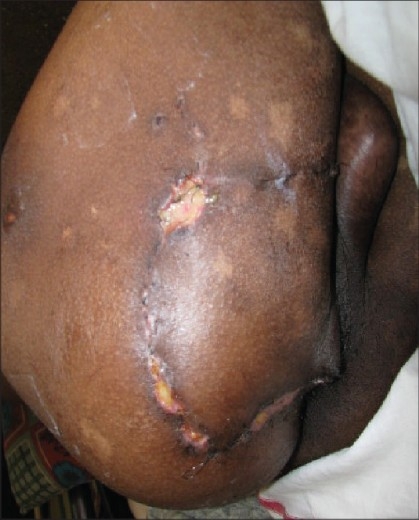
Twelfth postoperative day—minor dehiscence at one corner

We have used this flap in four cases of ischial pressure sore so far [Figures 1–4]. All the cases were post-traumatic permanent paraplegic patients. In all four cases the flap was based on the medial side. The ischial pressure sore is first thoroughly debrided. This includes excision of any fibrotic adventitious bursa over ischial tuberosity. Ischial tuberosity, if found infected may be shaved off without completely excising it. Radical excision of ischium should be avoided. Excising ischial tuberosity on one side leads to excessive weight being transmitted to the opposite side and may hasten occurrence of ischial pressure sore on the opposite side.[3] Excising the bony prominence may be advantageous in sacral and trochanteric pressure sores but not in ischial sore. The flap is marked on the skin just below the ischial defect. It should be of sufficient length and about three or four inches in width. Margins are incised down to the deep fascia. By blunt dissection superficial to the hamstring muscles the perforators entering the flap may be preserved. We saw two or three perforators in this area in each case. The perforators are quite long and do not restrict the movement of the flap. In one case there was a perforator near the tip of the flap which had to be divided before the flap could be transposed. The flap is about 1 to 1½ inches thick even in lean patients because of abundance of fat in this area and fills the sore nicely. If more padding is needed biceps femoris or any other hamstring muscle may be advanced over ischial tuberosity. After transposing the flap, the redundancy of skin below the gluteal fold allows tension-free closure of the secondary defect. This is a great advantage of this flap as split-skin graft-covered secondary defect is liable to ulcerate very easily within a short period of time. A suction drain is kept underneath the flap and brought out through a separate stab incision. Sutures and drainage tube may be removed after two weeks.

## RESULTS

In all four cases the flap survived fully. In one case there was a minor dehiscence of suture line at one corner due to infection possibly due to faecal contamination, but did not require any intervention and the wound healed in due course.

## DISCUSSION

A flap used for any pressure sore should ideally satisfy the following conditions.

1. The flap should be quite thick to fill the sore and also provide good padding over the bony prominence.
2. The flap should have reliable blood supply so that healing takes place by first intention.
3. The flap should not preclude usage of another flap in case of recurrence of pressure sore.
4. The flap should preferably not leave a secondary defect covered by split-skin graft.

The medial circumflex femoral artery flap satisfies all the above mentioned criteria. Moreover, the flap is technically easy to do. It can also be combined with biceps femoris muscle advancement if needed to provide more padding over ischial tuberosity.

## CONCLUSION

The medial circumflex femoral artery flap is the flap of first choice for ischial pressure sore. A study based on a much larger number of patients is required to further establish the same. The vascular basis of this flap, i.e. the terminal branches of medial circumflex femoral artery entering the deep fascia through the interval between quadratus femoris and adductor magnus is well established and is described in all standard textbooks of anatomy.
